# A critical period for paired-housing-dependent autistic-like behaviors attenuation in a prenatal valproic acid-induced male mouse model of autism

**DOI:** 10.3389/fnins.2024.1467047

**Published:** 2025-01-17

**Authors:** Jian-Quan Yang, Bao-Qi Yin, Chao-Hua Yang, Miao-Miao Jiang, Zhe Li

**Affiliations:** Department of Children's Rehabilitation, Research Center of Rehabilitation Medicine in Henan, The Fifth Affiliated Hospital of Zhengzhou University, Zhengzhou, Henan, China

**Keywords:** autism spectrum disorder, valproic acid, paired-housing, critical period, GABAergic neurotransmission

## Abstract

Autism spectrum disorder (ASD) is a neurodevelopmental disorder characterized by impairments in social communication and the presence of restrictive and repetitive behaviors. Investigating the etiological process and identifying an appropriate therapeutic target remain as formidable challenges to overcome ASD due to numerous risk factors and complex symptoms associated with the disorder. Recent studies have indicated that early rehabilitative intervention can alleviate the symptoms of individuals with ASD. However, there remain unsolved issues of behavioral intervention such as the appropriate time and types of therapies. In this study, we employed a mouse model prenatally exposed to valproic acid to establish a validated ASD mouse model and We found that paired-housing with control mice for 4 week after weaning palliated sociability deficits, anxiety and repetitive behaviors in this model of ASD-like behaviors, while paired-housing with their ASD littermate did not produce this effects. Furthermore, by evaluating different time window of paired-housing, we found that paired-housing during postnatal day 21 (P21) to P35, but not P21 to P28 or P35 to P49 or P28 to P35, is a critical period for the influence of paired-housing on autistic-like behaviors. Finally, paired-housing with control mice improved the impaired GABA system in this model of ASD. So our study demonstrates the therapeutic potential of environmental intervention during a critical period in the treatment of ASD.

## 1 Introduction

Autism spectrum disorder (ASD) is a neurodevelopmental disorder characterized by two core symptoms, namely impaired social interaction and communication, and repetitive behaviors (Silverman et al., 2010; Bourgeron, 2015). Despite a number of studies conducted to elucidate the remedy, there are few established treatments for symptom improvement. Recent studies, however, have indicated the effectiveness of rehabilitative interventions to alleviate the social problems of individuals with ASD, and substantial efforts have been devoted to the development of such rehabilitative interventions. The most common rehabilitative intervention is applied behavioral analysis, which is an intense behavioral intervention that improves intellectual and educational functioning (Foo et al., 2019; Lovaas, 1987). Several brain imaging studies using functional magnetic resonance imaging (fMRI) revealed that rehabilitative interventions, including computer-based facial affect recognition training, pivotal response treatment, and reading intervention, can improve ASD symptoms by affecting brain functions and connectivity (Calderoni et al., 2016; Bharathi et al., 2019).

Recent studies have broadened the molecular and clinical genetics as well as epidemiology of ASD, but the current therapeutic options for these children are still limited to the early behavioral interventions and there are unsolved issues of behavioral intervention such as the appropriate time and types of therapies (Calderoni et al., 2016; Bharathi et al., 2019). In clinical settings, prospective controlled approaches in a consistent environmental context are impossible owing to the great heterogeneity of the genetic and environmental features of patients with ASD as well as ethical problems, and most clinical studies are inadequate with short-term data from a heterogeneous group of patients (Zwaigenbaum et al., 2015; Woo and Leon, 2013).

It has been extensively shown that prenatal exposure of mice to valproic acid (VPA) at gestational day 12.5 results in reduced social interaction in the adult male off-spring and increased stereotyped behaviors (Ornoy, 2009; Tartaglione et al., 2019), and our previous study also found that prenatally VPA-exposed mice exhibited autistic-like behaviors (Yang et al., 2021), which was suitable to investigate mechanisms of ASD and its therapeutic strategy. In these studies, experimental mice exposed to VPA were weaned with their own littermates (Ornoy, 2009; Tartaglione et al., 2019; Yang et al., 2021). And recent study found that early social enrichment can specifically rescue social deficits in a prenatal valproic acid-induced mouse model of autism (Campolongo et al., 2018). However, the intervention of the autistic brain using other methods remains a challenge in neuroscience research.

Therefore, the aim of this study was to use the VPA-induced ASD mice model to evaluate the effects of a critical period for group-housing on social interaction deficits and recognition memory impairments in mice exposed prenatally to VPA. Moreover, since our previous study has demonstrated an intimate relationship between gamma-aminobutyric acid (GABA) receptors and ASD (Yang et al., 2021), we also studied whether these receptors were involved in this phenomenon.

## 2 Materials and methods

### 2.1 Animals

**Figure 1:**
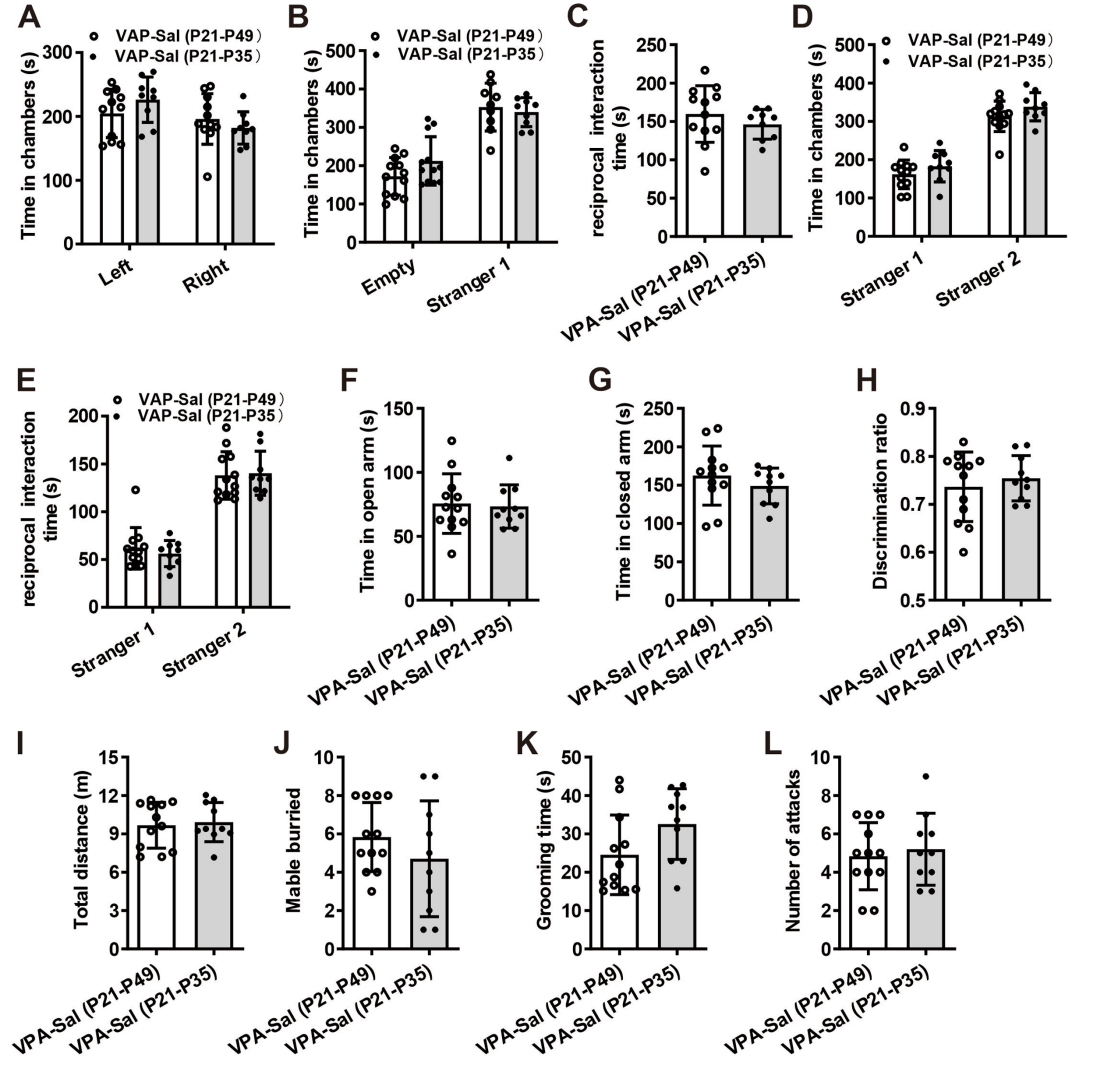
Prenatally VPA-exposed mice showed autistic-like behaviors. **(A)** Schematic of the experiments tests (upper) and the experimental timeline of behavioral tests (down). **(B–F)** Impaired social interaction in prenatally VPA-exposed mice in the three-chamber test (*n* = 12 mice/group; two-tailed Student’s t-test; for **(B)** Ctrl group, *t*_(22)_ = 0.432, *p* = 0.734; VPA group, *t*_(22)_ = 0.386, *p* = 0.628; for **(C)** Ctrl group, *t*_(22)_ = 3.534, *p* = 0.007; VPA group, *t*_(22)_ = 0.963, *p* = 0.273; for **(D)** *t*_(22)_ = 5.743, *p* < 0.001; for **(E)** Ctrl group, *t*_(22)_ = 3.265, *p* = 0.032; VPA group, *t*_(22)_ = 0.735, *p* = 0.408; for **(F)** Ctrl group, *t*_(22)_ = 3.063, *p* = 0.004; VPA group, *t*_(22)_ = 0.496, *p* = 0.473). **(G,H)** Prenatally VPA-exposed mice exhibited anxiety-like behavior in the EPM (*n* = 12 mice/group; two-tailed Student’s t-test; for **(G)** *t*_(22)_ = 4.024, *p* = 0.005; for **(H)** *t*_(22)_ = 3.589, *p* = 0.012). **(I)** Impaired recognition memory in prenatally VPA-exposed mice in the NOR test (*n* = 12 mice/group; two-tailed Student’s t-test; *t*_(22)_ = 3.571, *p* = 0.013). **(J)** The locomotor activity in the open field test was not affected between two groups (*n* = 12 mice/group; two-tailed Student’s t-test; *t*_(22)_ = 0.785, *p* = 0.672). **(K,L)** Increased repetitive behavior in prenatally VPA-exposed mice in the marble-burying test **(K)** and the self-grooming test **(L)** (*n* = 12 mice/group; two-tailed Student’s t-test; for **(K)** *t*_(22)_ = 3.467, *p* = 0.013; for **(L)** *t*_(22)_ = 5.854, *p* = 0.0002). Data are presented as the mean ± s.e.m. *p < 0 0.05, **p < 0.01, ***p < 0.001.

**Figure 2:**
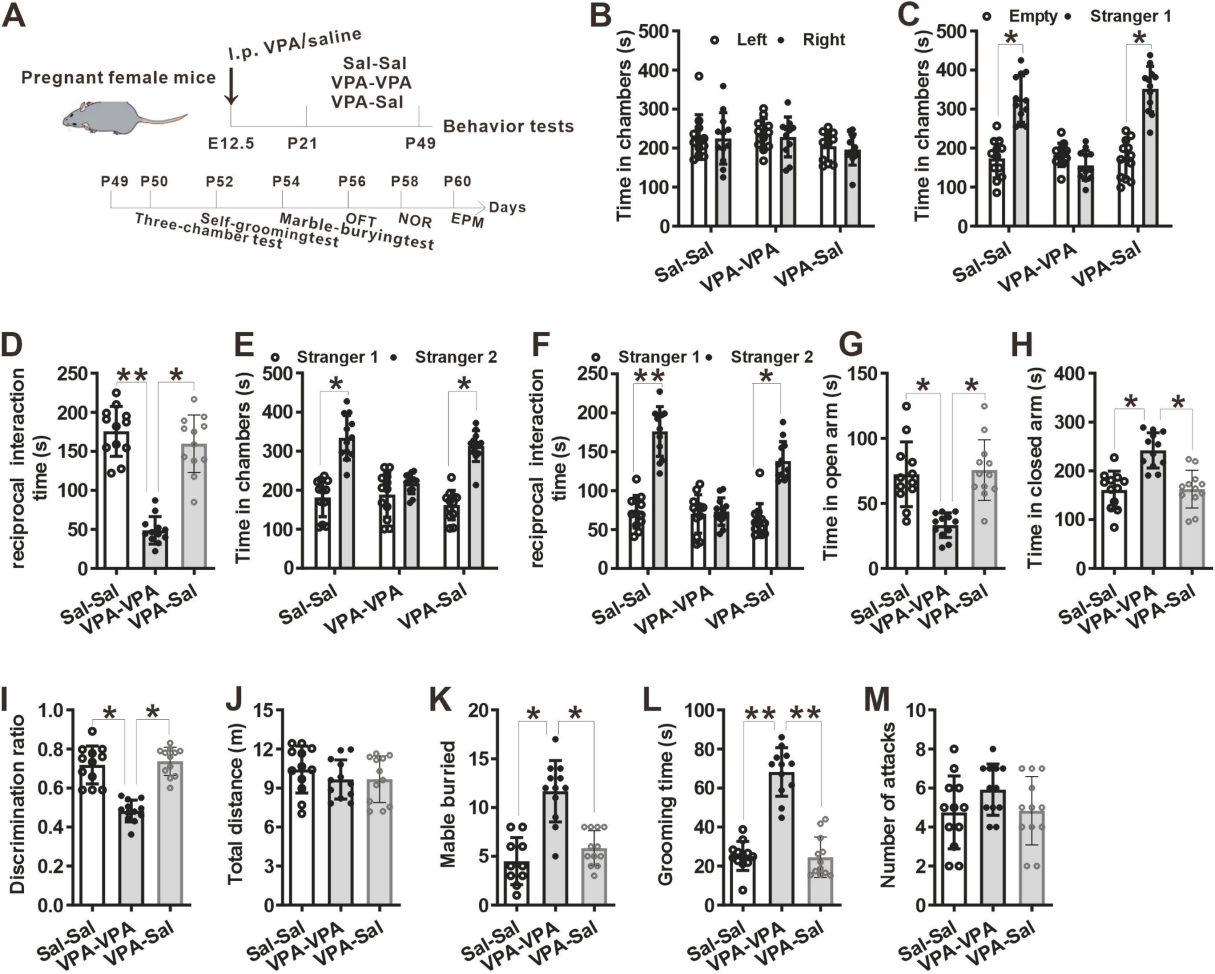
Four-week paired-housing with control mice rescued autistic-like behaviors in prenatally VPA-exposed mice. **(A)** Schematic of the experiments tests (upper) and the experimental timeline of behavioral tests (down). **(B–F)** Four-week paired-housing with control mice rescued the impaired social interaction in prenatally VPA-exposed mice in the three-chamber test (*n* = 12 mice/group; two-way ANOVA with “prenatal treatment” and “group-housing environment” interaction; for **(B)** *F*_(2, 33)_ = 0.636, *p* = 0.922; for **(C)** *F*_(2, 33)_ = 4.764, *p* = 0.043; for **(D)** *F*_(2, 33)_ = 5.024, *p* = 0.021; for **(E)** *F*_(2, 33)_ = 4.702, *p* = 0.045; for **(F)** *F*_(2, 33)_ = 5.834, *p* = 0.015). **(G,H)** Prenatally VPA-exposed mice exhibited anxiety-like behavior in the EPM, which was rescued by paired-housing with control mice for 4 weeks (*n* = 12 mice/group; two-way ANOVA with “prenatal treatment” and “group-housing environment” interaction; for **(G)** *F*_(2, 33)_ = 6.16, *p* = 0.031; for **(H)** *F*_(2, 33)_ = 5.79, *p* = 0.037). **(I)** Impaired recognition memory in prenatally VPA-exposed mice was rescued by paired-housing treatment in the NOR test (*n* = 12 mice/group; two-way ANOVA with “prenatal treatment” and “group-housing environment” interaction; *F*_(2, 33)_ = 5.25, *p* = 0.026). **(J)** The locomotor activity in the open field test was not affected between these groups (*n* = 12 mice/group; two-way ANOVA with “prenatal treatment” and “group-housing environment” interaction; *F*_(2, 33)_ = 2.08, *p* = 0.703). **(K,L)** Increased repetitive behavior in prenatally VPA-exposed mice was rescued by four-week paired-housing with control mice in the marble-burying test **(K)** and the self-grooming test **(L)** (*n* = 12 mice/group; two-way ANOVA with “prenatal treatment” and “group-housing environment” interaction; for **(K)** *F*_(2, 33)_ = 4.35, *p* = 0.044; for **(L)** *F*_(2, 33)_ = 5.04, *p* = 0.037). **(M)** Four-week paired-housing with other mouse did not affect the attack behaviors (*n* = 12 mice/group; two-way ANOVA with “prenatal treatment” and “group-housing environment” interaction; *F*_(2, 33)_ = 1.85, *p* = 0.326). Data are presented as the mean ± s.e.m. *p < 0 0.05, **p < 0.01.

**Figure 3:**
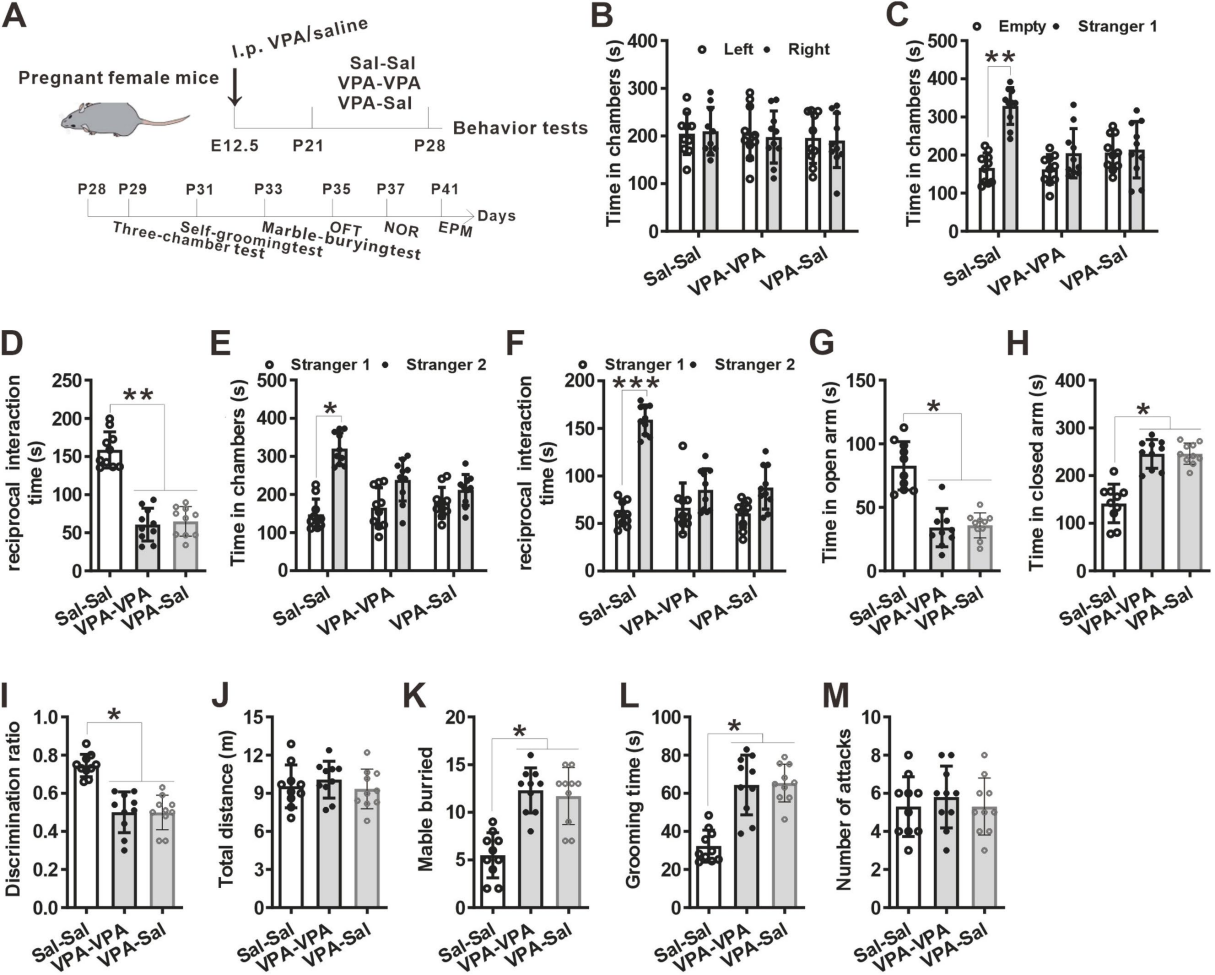
One-week paired-housing with control mice had no effect on autistic-like behaviors in prenatally VPA-exposed mice. **(A)** Schematic of the experiments tests (upper) and the experimental timeline of behavioral tests (down). **(B–F)** One-week paired-housing with control mice did not rescue the impaired social interaction in prenatally VPA-exposed mice in the three-chamber test (*n* = 10 mice/group; two-way ANOVA with “prenatal treatment” and “group-housing environment” interaction; for **(B)** *F*_(2, 27)_ = 0.474, *p* = 0.957; for **(C)** *F*_(2, 27)_ = 4.086, *p* = 0.042; for **(D)** *F*_(2, 27)_ = 4.257, *p* = 0.041; for **(E)** *F*_(2, 27)_ = 4.046, *p* = 0.047; for **(F)** *F*_(2, 27)_ = 5.357, *p* = 0.034). **(G,H)** Prenatally VPA-exposed mice exhibited anxiety-like behavior in the EPM, which was not rescued by paired-housing with control mice for 1 week (*n* = 10 mice/group; two-way ANOVA with “prenatal treatment” and “group-housing environment” interaction; for **(G)** *F*_(2, 27)_ = 4.36, *p* = 0.031; for **(H)** *F*_(2, 33)_ = 5.01, *p* = 0.033). **(I)** Impaired recognition memory in prenatally VPA-exposed mice was not rescued by paired-housing treatment in the NOR test (*n* = 10 mice/group; two-way ANOVA with “prenatal treatment” and “group-housing environment” interaction; *F*_(2, 27)_ = 4.45, *p* = 0.036). **(J)** The locomotor activity in the open field test was not affected between these groups (*n* = 10 mice/group; two-way ANOVA with “prenatal treatment” and “group-housing environment” interaction; *F*_(2, 27)_ = 2.09, *p* = 0.417). **(K,L)** Increased repetitive behavior in prenatally VPA-exposed mice was not rescued by one-week paired-housing with control mice in the marble-burying test **(K)** and the self-grooming test **(L)** (*n* = 10 mice/group; two-way ANOVA with “prenatal treatment” and “group-housing environment” interaction; for **(K)** *F*_(2, 27)_ = 4.65, *p* = 0.035; for **(L)** *F*_(2, 33)_ = 5.23, *p* = 0.033). **(M)** Paired-housing with other mouse from P21 to P28 did not affect the attack behaviors (*n* = 10 mice/group; two-way ANOVA with “prenatal treatment” and “group-housing environment” interaction; *F*_(2, 27)_ = 2.23, *p* = 0.209). Data were presented as mean ± s.e.m. *p < 0 0.05, **p < 0.01.

**Figure 4:**
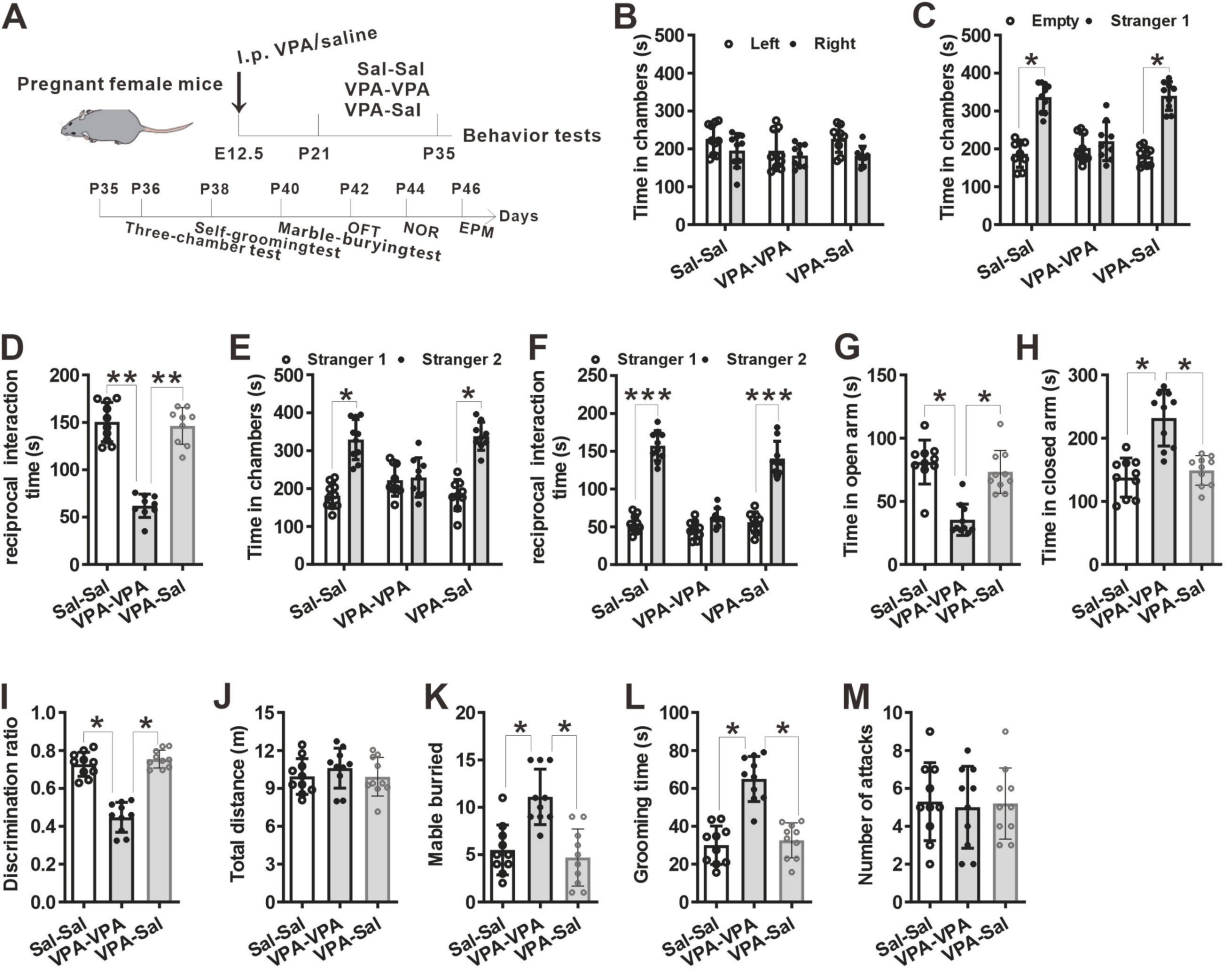
The effects of paired-housing with control mice on autistic-like behaviors occurred only during a critical period. **(A)** Schematic of the experiments tests (upper) and the experimental timeline of behavioral tests (down). **(B–F)** Two-week paired-housing with control mice rescued the impaired social interaction in prenatally VPA-exposed mice in the three-chamber test (*n* = 10 mice/group; two-way ANOVA with “prenatal treatment” and “group-housing environment” interaction; for **(B)** *F*_(2, 27)_ = 0.574, *p* = 0.843; for **(C)** *F*_(2, 27)_ = 4.743, *p* = 0.031; for **(D)** *F*_(2, 27)_ = 5.743, *p* = 0.027; for **(E)** *F*_(2, 27)_ = 4.432, *p* = 0.038; for **(F)** *F*_(2, 27)_ = 5.987, *p* = 0.018). **(G,H)** Prenatally VPA-exposed mice exhibited anxiety-like behavior in the EPM, which was rescued by paired-housing with control mice for 2 weeks (*n* = 10 mice/group; two-way ANOVA with “prenatal treatment” and “group-housing environment” interaction; for **(G)** *F*_(2, 27)_ = 4.46, *p* = 0.027; for **(H)** *F*_(2, 33)_ = 4.76, *p* = 0.038). **(I)** Impaired recognition memory in prenatally VPA-exposed mice was rescued by paired-housing treatment in the NOR test (*n* = 10 mice/group; two-way ANOVA with “prenatal treatment” and “group-housing environment” interaction; *F*_(2, 27)_ = 4.09, *p* = 0.039). **(J)** The locomotor activity in the open field test was not affected between these groups (*n* = 10 mice/group; two-way ANOVA with “prenatal treatment” and “group-housing environment” interaction; *F*_(2, 27)_ = 1.25, *p* = 0.472). **(K,L)** Increased repetitive behavior in prenatally VPA-exposed mice was rescued by two-week paired-housing with control mice in the marble-burying test **(K)** and the self-grooming test **(L)** (*n* = 10 mice/group; two-way ANOVA with “prenatal treatment” and “group-housing environment” interaction; for **(K)** *F*_(2, 27)_ = 4.87, *p* = 0.034; for **(L)** *F*_(2, 33)_ = 5.02, *p* = 0.036). **(M)** Paired-housing with other mouse from P21 to P35 did not affect the attack behaviors (*n* = 10 mice/group; two-way ANOVA with “prenatal treatment” and “group-housing environment” interaction; *F*_(2, 27)_ = 1.58, *p* = 0.257). Data were presented as mean ± s.e.m. *p < 0 0.05, **p < 0.01, ***p < 0.001.

**Figure 5:**
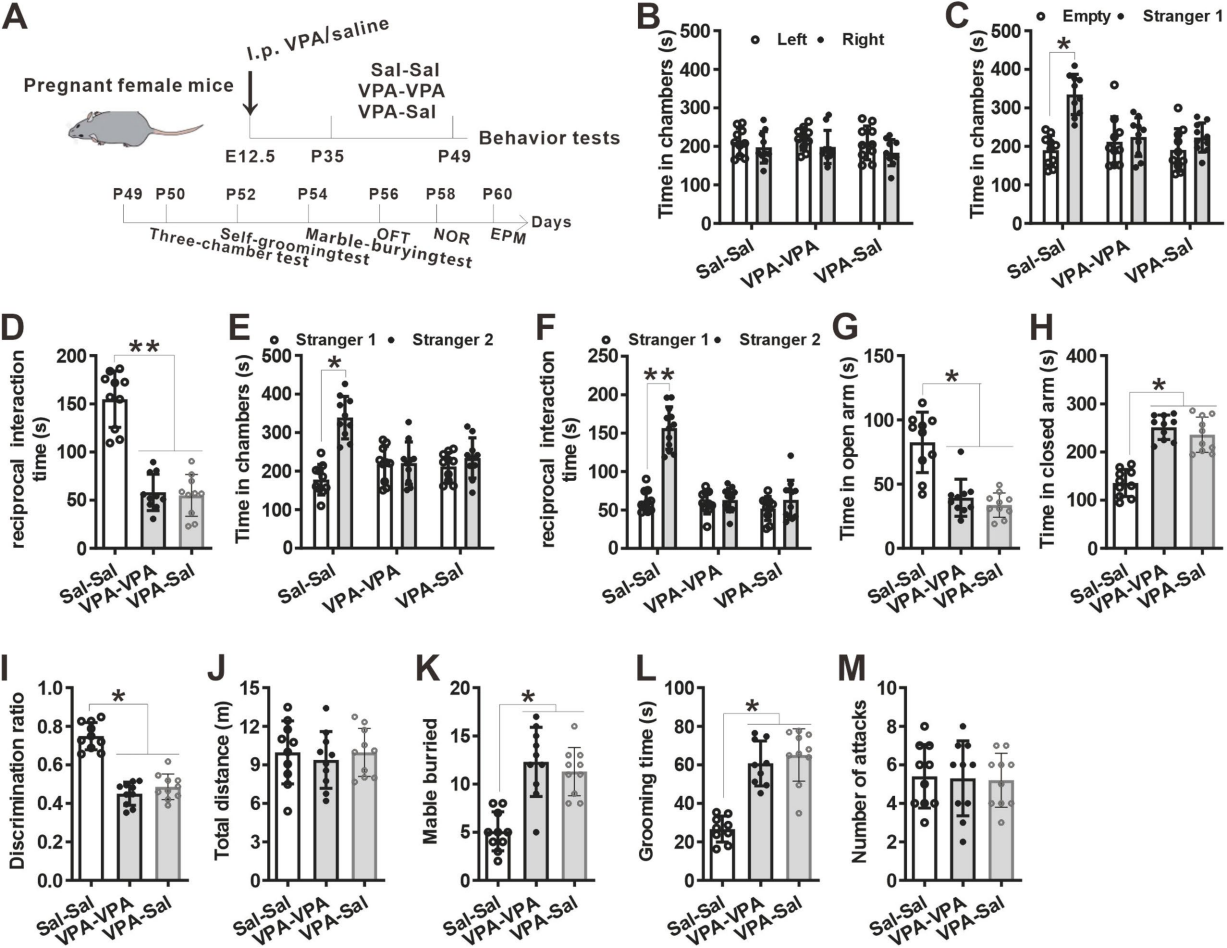
Two-week paired-housing with control mice begun at P35 had no effect on autistic-like behaviors in prenatally VPA-exposed mice. **(A)** Schematic of the experiments tests (upper) and the experimental timeline of behavioral tests (down). **(B–F)** Two-week paired-housing with control mice begun at P35 did not rescue the impaired social interaction in prenatally VPA-exposed mice in the three-chamber test (*n* = 10 mice/group; two-way ANOVA with “prenatal treatment” and “group-housing environment” interaction; for **(B)** *F*_(2, 27)_ = 0.257, *p* = 0.975; for **(C)** *F*_(2, 27)_ = 4.09, *p* = 0.046; for **(D)** *F*_(2, 27)_ = 5.57, *p* = 0.027; for **(E)** *F*_(2, 27)_ = 4.11, *p* = 0.044; for **(F)** *F*_(2, 27)_ = 4.77, *p* = 0.038). **(G,H)** Prenatally VPA-exposed mice exhibited anxiety-like behavior in the EPM, which was not rescued by paired-housing with control mice for 2 weeks begun at P35 (*n* = 10 mice/group; two-way ANOVA with “prenatal treatment” and “group-housing environment” interaction; for **(G)** *F*_(2, 27)_ = 4.09, *p* = 0.039; for **(H)** *F*_(2, 33)_ = 4.22, *p* = 0.035). **(I)** Impaired recognition memory in prenatally VPA-exposed mice was not rescued by paired-housing treatment in the NOR test (*n* = 10 mice/group; two-way ANOVA with “prenatal treatment” and “group-housing environment” interaction; *F*_(2, 27)_ = 4.12, *p* = 0.038). **(J)** The locomotor activity in the open field test was not affected between these groups (*n* = 10 mice/group; two-way ANOVA with “prenatal treatment” and “group-housing environment” interaction; *F*_(2, 27)_ = 1.09, *p* = 0.653). **(K,L)** Increased repetitive behavior in prenatally VPA-exposed mice was not rescued by two-week paired-housing with control mice begun at P35 in the marble-burying test **(K)** and the self-grooming test **(L)** (*n* = 10 mice/group; two-way ANOVA with “prenatal treatment” and “group-housing environment” interaction; for **(K)** *F*_(2, 27)_ = 4.78, *p* = 0.035; for **(L)** *F*_(2, 33)_ = 4.88, *p* = 0.039). **(M)** Paired-housing with other mouse from P35 to P49 did not affect the attack behaviors (*n* = 10 mice/group; two-way ANOVA with “prenatal treatment” and “group-housing environment” interaction; *F*_(2, 27)_ = 1.32, *p* = 0.298). Data were presented as mean ± s.e.m. *p < 0 0.05, **p < 0.01.

**Figure 6:**
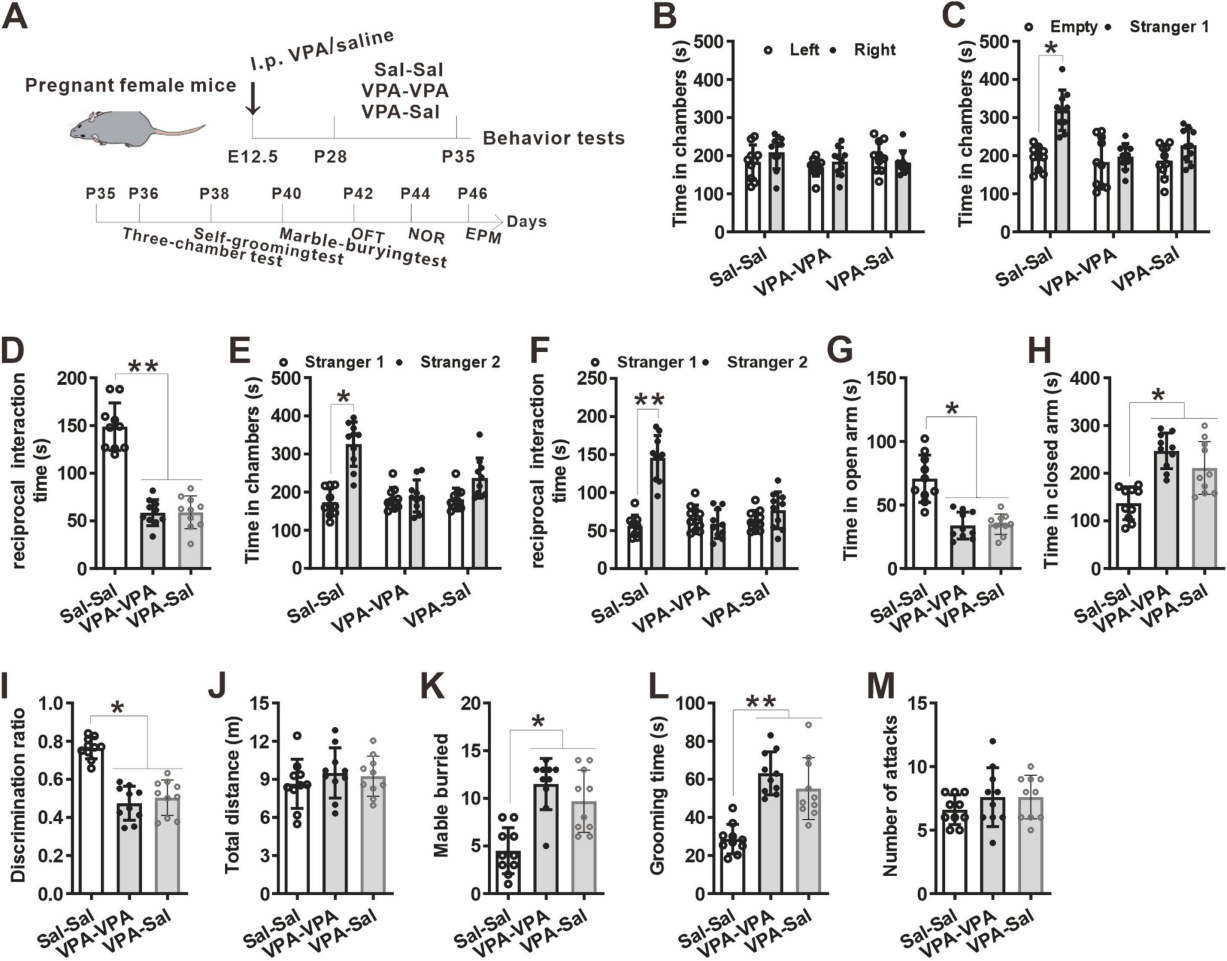
One-week paired-housing with control mice begun at P28 had no effect on autistic-like behaviors in prenatally VPA-exposed mice. **(A)** Schematic of the experiments tests(upper) and the experimental timeline of behavioral tests (down). **(B–F)** One-week paired-housing with control mice begun at P28 did not rescue the impaired social interaction in prenatally VPA-exposed mice in the three-chamber test (*n* = 10 mice/group; two-way ANOVA with “prenatal treatment” and “group-housing environment” interaction; for **(B)** *F*_(2, 27)_ = 0.633, *p* = 0.742; for **(C)** *F*_(2, 27)_ = 4.32, *p* = 0.044; for **(D)** *F*_(2, 27)_ = 5.75, *p* = 0.023; for **(E)** *F*_(2, 27)_ = 4.43, *p* = 0.040; for **(F)** *F*_(2, 27)_ = 4.96, *p* = 0.033). **(G,H)** Prenatally VPA-exposed mice exhibited anxiety-like behavior in the EPM, which was not rescued by paired-housing with control mice for 1 week begun at P28 (*n* = 10 mice/group; two-way ANOVA with “prenatal treatment” and “group-housing environment” interaction; for **(G)** *F*_(2, 27)_ = 4.43, *p* = 0.036; for **(H)** *F*_(2, 33)_ = 4.47, *p* = 0.030). **(I)** Impaired recognition memory in prenatally VPA-exposed mice was not rescued by paired-housing treatment in the NOR test (*n* = 10 mice/group; two-way ANOVA with “prenatal treatment” and “group-housing environment” interaction; *F*_(2, 27)_ = 4.42, *p* = 0.033). **(J)** The locomotor activity in the open field test was not affected between these groups (*n* = 10 mice/group; two-way ANOVA with “prenatal treatment” and “group-housing environment” interaction; *F*_(2, 27)_ = 1.24, *p* = 0.527). **(K,L)** Increased repetitive behavior in prenatally VPA-exposed mice was not rescued by one-week paired-housing with control mice begun at P28 in the marble-burying test **(K)** and the self-grooming test **(L)** (*n* = 10 mice/group; two-way ANOVA with “prenatal treatment” and “group-housing environment” interaction; for **(K)** *F*_(2, 27)_ = 4.97, *p* = 0.031; for **(L)** *F*_(2, 33)_ = 5.13, *p* = 0.027). **(M)** Paired-housing with other mouse from P28 to P35 did not affect the attack behaviors (*n* = 10 mice/group; two-way ANOVA with “prenatal treatment” and “group-housing environment” interaction; *F*_(2, 27)_ = 1.08, *p* = 0.416). Data were presented as mean ± s.e.m. *p < 0 0.05, **p < 0.01.

**Figure 7:**
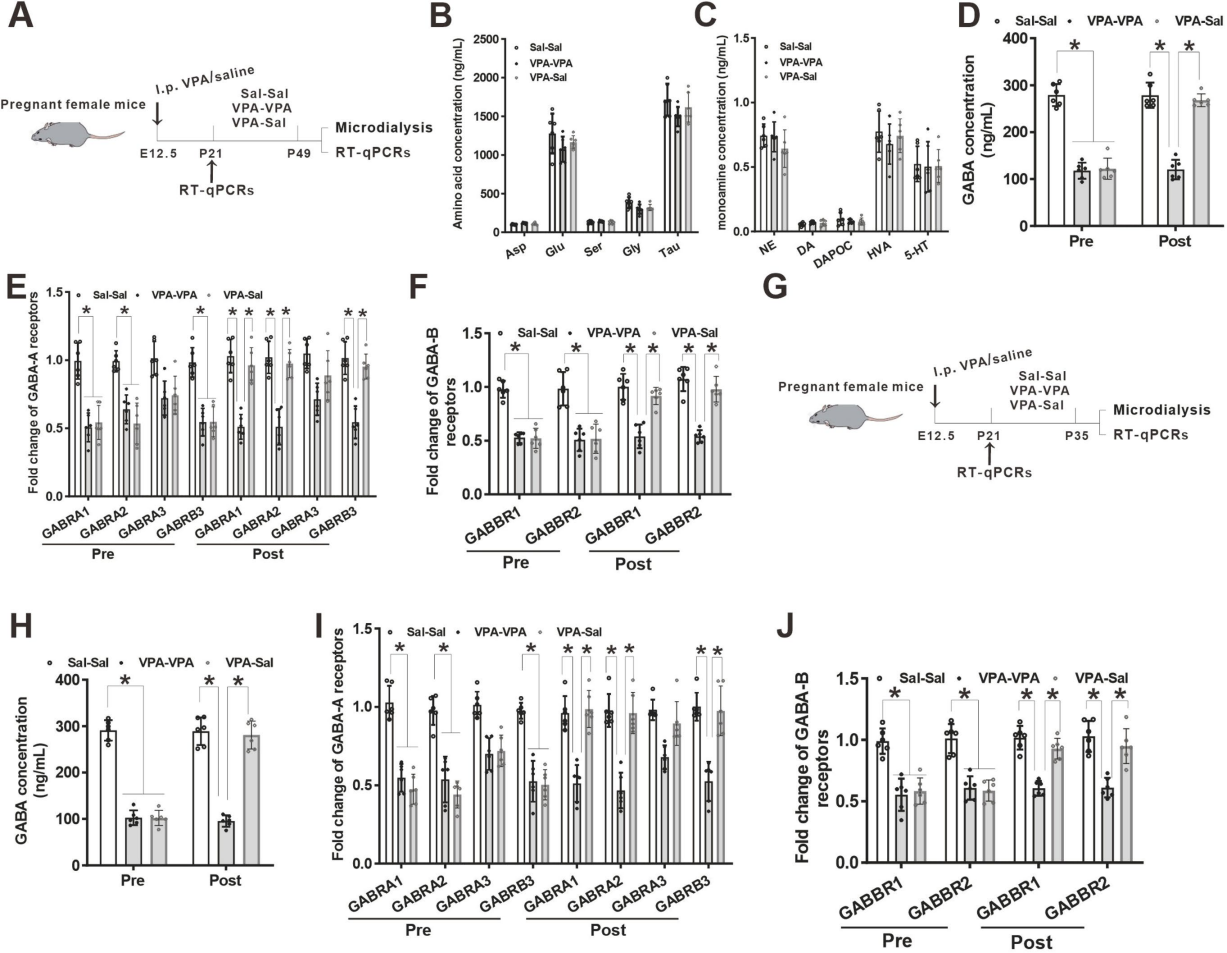
Paired-housing with control mice rescued GABA level and GABA receptors reduction in prenatally VPA-exposed mice. **(A)** Schematic of the experiments tests (P21–P49). **(B)** Microdialysis measurements of amino acids concentration in the mPFC region from paired-housing mice from P21 to P49 (*n* = 6 mice/group; two-way ANOVA with “prenatal treatment” and “group-housing environment” interaction; Asp: aspartic acid, *F*_(2,15)_ = 2.43, *p* = 0.421; Glu: glutamate, *F*_(2, 15)_ = 1.74, *p* = 0.476; Ser: serine, *F*_(2, 15)_ = 1.42, *p* = 0.184; Gly: glycine, *F*_(2, 15)_ = 2.54, *p* = 0.325; Tau: taurine, *F*_(2, 15)_ = 1.78, *p* = 0.547). **(C)** Microdialysis measurements of monoamines concentration in the mPFC region from P21 to P49 paired-housing mice (*n* = 6 mice/group; two-way ANOVA with “prenatal treatment” and “group-housing environment” interaction; NE: norepinephrine, *F*_(2, 15)_ = 1.75, *p* = 0.462; DA: dopamine, *F*_(2, 15)_ = 2.34, *p* = 0.346; DAPOC: 3,4-Dihydroxybenzoic acid, a metabolin of DA, *F*_(2, 15)_ = 2.64, *p* = 0.356; HVA: 4-hydroxy-3-methoxyphenylacetic, another metabolin of DA, *F*_(2, 15)_ = 2.76, *p* = 0.509; 5-HT: serotonin, *F*_(2, 15)_ = 2.87, *p* = 0.206). **(D)** Microdialysis measurements of GABA concentration in the mPFC region from ASD model mice before and after P21–P49 paired-housing (*n* = 6 mice/group; two-way ANOVA with “prenatal treatment” and “group-housing environment” interaction; GABA: γ-aminobutyric acid, Pre: *F*_(2, 15)_ = 4.36, *p* = 0.032; Post: *F*_(2, 15)_ = 3.98, *p* = 0.037). **(E)** Q-PCR measurements of GABA-A receptor subunits level in the mPFC region before and after P21–P49 paired-housing mice (*n* = 6/group; two-way ANOVA with “prenatal treatment” and “group-housing environment” interaction; GABRA1, Pre: *F*_(2, 15)_ = 4.75, *p* = 0.027; Post: *F*_(2, 15)_ = 4.95, *p* = 0.022; GABRA2, Pre: *F*_(2, 15)_ = 4.95, *p* = 0.024; Post: *F*_(2, 15)_ = 5.37, *p* = 0.020; GABRA3, Pre: *F*_(2, 15)_ = 3.43, *p* = 0.097; Post: *F*_(2, 15)_ = 3.87, *p* = 0.088; GABRB3, Pre: *F*_(2, 15)_ = 4.76, *p* = 0.035; Post: *F*_(2, 15)_ = 4.99, *p* = 0.031). **(F)** Q-PCR measurements of GABA-B receptor subunits level in the mPFC region before and after P21–P49 paired-housing mice (*n* = 6/group; two-way ANOVA with “prenatal treatment” and “group-housing environment” interaction; GABBR1, Pre: *F*_(2, 15)_ = 4.89, *p* = 0.022; Post: *F*_(2, 15)_ = 4.98, *p* = 0.019; GABBR2, Pre: *F*_(2, 15)_ = 4.77, *p* = 0.025; Post: *F*_(2, 15)_ = 4.87, *p* = 0.022). **(G)** Schematic of the experiments tests (P21–P35). **(H)** Microdialysis measurements of GABA concentration in the mPFC region from ASD model mice before and after P21–P35 paired-housing (*n* = 6 mice/group; two-way ANOVA with “prenatal treatment” and “group-housing environment” interaction; Pre: *F*_(2, 15)_ = 4.12, *p* = 0.039; Post: *F*_(2, 15)_ = 3.43, *p* = 0.041). **(I)** Q-PCR measurements of GABA-A receptor subunits level in the mPFC region before and after P21–P35 paired-housing mice (*n* = 6/group; two-way ANOVA with “prenatal treatment” and “group-housing environment” interaction; GABRA1, Pre: *F*_(2, 15)_ = 4.46, *p* = 0.033; Post: *F*_(2, 15)_ = 4.56, *p* = 0.029; GABRA2, Pre: *F*_(2, 15)_ = 4.48, *p* = 0.028; Post: *F*_(2, 15)_ = 5.02, *p* = 0.026; GABRA3, Pre: *F*_(2, 15)_ = 3.04, *p* = 0.159; Post: *F*_(2, 15)_ = 3.34, *p* = 0.102; GABRB3, Pre: *F*_(2, 15)_ = 4.58, *p* = 0.038; Post: *F*_(2, 15)_ = 4.68, *p* = 0.034). **(J)** Q-PCR measurements of GABA-B receptor subunits level in the mPFC region before and after P21–P35 paired-housing mice (*n* = 6/group; two-way ANOVA with “prenatal treatment” and “group-housing environment” interaction; GABBR1, Pre: *F*_(2, 15)_ = 4.72, *p* = 0.025; Post: *F*_(2, 15)_ = 4.88, *p* = 0.021; GABBR2, Pre: *F*_(2, 15)_ = 4.54, *p* = 0.028; Post: *F*_(2, 15)_ = 4.67, *p* = 0.024). Data were presented as mean ± s.e.m. *p < 0.05.

Male and female C57BL/6J mice were obtained from the Laboratory Animal Center of Zhengzhou University (Zhengzhou, China) at 8 weeks of age and mated in harem style. Pregnancy was validated by the observation of a vaginal plug on embryonic day 0 (E0), then the male mice were removed from the cage. The animals were kept in standard laboratory cages under a 12-h light/dark cycle (with lights on at 8:00 A.M.) in a temperature-controlled environment (21–25°C), and were provided with ad libitum access to food (D12450B, Research Diets, New Brunswick, NJ, United States) and water. On E12.5, 17 pregnant females were intraperitoneally (i.p.) injected with 500 mg/kg VPA (Sigma-Aldrich, St. Louis, MO, USA) dissolved in isotonic 0.9% sodium chloride solution, and another 15 pregnant females received an equal volume of saline. Pups from VPA-exposed mothers (Group 1) and from mothers that received saline (Group 2) were used in the study after weaning at 3 weeks of age. In behavior detection, sexual dimorphisms occur. And the emergence of menstruation in female mice is a contributory factor in the behavioral test. We totally obtained 168 male offspring from 17 VPA-exposed mothers (Group 1) and 172 male offspring from 15 mothers that received saline (Group 2) were divided into two subgroups as shown in the schematic experimental design (Figure 1), three subgroups in Figure 2, three subgroups in Figure 3, three subgroups in Figure 4, three subgroups in Figure 5, three subgroups in Figure 6 and further for biochemical experiment in Figure 7. All procedures were conducted in accordance with the Chinese Council on Animal Care Guidelines, and efforts were made to minimize animal suffering and to reduce the number of animals used.

### 2.2 Behavioral tests

A discrete room within the animal facility, maintained at 22°C with 40–70% humidity, was designated for behavioral tests to minimize external disturbances. Diffuse reflection light source was employed to ensure a subdued lighting environment. Mice were allowed to acclimate to the testing room for 1 h before the experiments. All the experiments were performed from 13:00 to 18:00, unless otherwise indicated. Sequentially, mice underwent three-chamber test, self-grooming test, marble-burying test, open field test, novel object recognition (NOR) test, and elevated plus-maze (EPM) test. Silence was maintained during the tests. After each round of testing, the apparatus used was wiped with 75% ethanol to remove any residual odors from mice. The study design is illustrated in each figure. In our study, after 1 week or 2 weeks or 4 weeks of group housing with control mice, the VPA-exposed animals were single-housed during the next behavioral tests.

### 2.3 Three-chamber test

The three-chamber test was used to assess social approach and social novelty preference that was conducted as described previously (Yang et al., 2021). The apparatus used was a rectangular box divided into three chambers, measuring 60 cm × 40 cm (Zhengzhou Baocheng, Henan, China). Following a 10-min habituation in the central chamber, each mouse was permitted to explore all of the empty chambers for an additional 10 min. Next, an unfamiliar C57BL/6J mouse (designated as stranger 1), confined within a wire cage, was introduced into one of the two side chambers while an identical empty wire cage (10.5 cm diameter, Galaxy Pencil Cup, Spectrum Diversified Designs) was placed in another side chamber. Once the mouse was positioned in the center chamber, the side doors were opened, allowing the mouse to explore the environment for 10 min. Then, a new unfamiliar age- and gender-matched C57BL/6J stranger mouse (stranger 2) was placed in the empty wire cage, and the test mouse was observed for an additional 10 min. In this period, social novelty preference was assessed. The stranger mice were not been habituation to the wire cage before actual testing began. The time spent in each side chamber was recorded in an automated manner. And time spent in reciprocal interactions, such as nose-to-nose sniffing, anogenital sniffing, and grooming were also measured in an automated manner.

### 2.4 Self-grooming test

The self-grooming test was conducted as described previously (Yang et al., 2021). Each mouse was placed individually in a new Plexiglas cage (30 × 30 × 35 cm) and allowed to habituate to the empty environment for 10 min. Following habituation, the total time spent grooming all body regions during the subsequent 10-min period was recorded by EthoVision 11.0 software. Grooming included all sequences of face-wiping, scratching/rubbing of head and ears, and full-body grooming.

### 2.5 Marble-burying test

The marble-burying test was conducted as described previously (Yang et al., 2021). Mice were placed individually in a Plexiglas cage (40 × 40 × 30 cm) with 5 cm of fresh bedding using the Nesting materials. Twenty black marbles (14 cm in diameter) were prearranged in the cage in four evenly spaced rows of five marbles each. The task lasted for 30 min, after which the number of buried marbles was recorded. A marble was deemed to be buried if more than half of its surface was covered by bedding.

### 2.6 Elevated plus-maze (EPM) test

The elevated plus-maze test was conducted as described previously using a customized four-armed apparatus (Yang et al., 2021), which consisted of four arms (30 × 5 cm) with two open arms without walls and two closed arms with 15 × 25-cm high walls and was elevated 65 cm off the floor. Each mouse was placed in the center of the elevated plus-maze facing an open arm. Over 5 min, the time spent in each arm was recorded by EthoVision 11.0 software.

### 2.7 Open field test

The open field test was conducted as described previously (Yang et al., 2021). The open field apparatus consisted of a rectangular chamber (40 × 40 × 30 cm) constructed from gray polyvinyl chloride. Each mouse was carefully placed at the center of the chamber and allowed to move freely for 5 min, during which its behavior was tracked using an automated video monitoring system. A digitized image of the mouse’s movement path was captured and subsequently analyzed using EthoVision 11.0 software.

### 2.8 Novel object recognition (NOR) test

The NOR test was conducted as described previously (Yang et al., 2021). The apparatus was a rectangular chamber (33 × 33 × 20 cm) constructed from black polyvinyl chloride. Each mouse underwent a 5-min habituation session in the empty arena 1 day prior to the familiarization phase. During the familiarization session, two identical objects [a tower of Lego bricks (8-cm high and 3.2-cm wide, built in white, blue, yellow, red and green bricks) and a Falcon tissue culture flask filled with sand (9.5 cm high, 2.5 cm deep and 5.5 cm wide, transparent plastic with a blue bottle cap)] were placed 5 cm away from the walls, and mice were allowed to freely explore each object until they had accumulated 20 s of total exploration time or until the 10-min period had elapsed. Twenty-four hours later, one familiar object and one novel object (a ball 5.5 cm in diameter) were sequentially placed in the arena. The experiment ended when the mice reached 20 s of exploration for both objects or when the 10-min period expired. To assess the recognition memory of each mouse, the discrimination ratio was calculated. This ratio was determined by dividing the time spent exploring the novel object by the total exploration time.

### 2.9 Microdialysis

Mice were anesthetized and prepared for stereotaxic surgery (Stoelting). A guide cannula (CMA/7, CMA) was implanted into the medial prefrontal cortex (mPFC). The dummy was removed prior to the insertion of the microdialysis probe. The microdialysis probe (CMA/7, CMA) was then inserted through the guide cannula and connected to a CMA microinjection pump (CMA 402). Artificial cerebrospinal fluid (ASCF) was continuously perfused through the microdialysis probe at a constant rate of 1 μL/min. Samples were automatically collected using a microfraction collector (CMA 142).

### 2.10 Quantitative real-time PCR

Brain tissue samples were dissected and prepared following selection. The samples were immediately stored in TRIzol (Invitrogen) for RNA extraction, following the manufacturer’s instructions. Genomic DNA was removed using a gDNA eraser treatment (Takara), and 1.0 μg of RNA was used for the synthesis of first-strand complementary DNA (cDNA) (Takara). Quantitative reverse transcription PCR (qRT-PCR) was performed on a Stratagene Mx3000P thermal cycler, using the Universal qRT-PCR master mix for the target genes (Takara). The primers were designed and synthesized as detailed below:

**Table 1:** 

Gene	sense	antisense
GABRA1	GAGTCGTCCAATCCAGCAC	AGCCAGAAGGAAACCTGTGA
GABRA2	CGAACTACGCTCCCAACATC	CATGCGGTCGATCTTACTCA
GABRA3	CGAGTCAGCAAAGTGGACAA	TATGAGGGTTGGACCTCCTG
GABRB3	GAGGTCCTCACCTGCAGAAG	GTTTCCATGAGCATCCACCT
GABBR1	CTCATCCACCACGACAGCAAG	GAGCCGTAGGAAAGCACAATG
GABBR2	CAGAAAGTCGCTTCCCAAAG	TGCGGTTGCTCACAAAGTAG
GAPDH	ATCACCATCTTCCAGGAGCGA	AGTTGTCATGGATGACCTTGGC

### 2.11 Statistical analyses

The experimental data were analyzed using a *t*-test to compare the means of two independent groups, and two-way ANOVA with Bonferroni’s post-hoc test was applied for comparisons among multiple groups. All statistical analyses were performed using SPSS 22.0 software. The number of experimental animals is denoted by ‘*n*’. Data are presented as the mean ± standard error of the mean (SEM), unless otherwise specified in the text or figures. Statistical significance was set at *p* < 0.05, and GraphPad Prism 6.0 was used to generate the graphs.

## 3 Results

### 3.1 Paired-housing with control mice for 4 week attenuated autistic-like behaviors in prenatally VPA-exposed mice

Firstly, to verify if prenatally exposed to valproic acid can establish a validated ASD mouse model, we examined behavioral changes in VPA-exposed mice (VPA group) and saline-exposed mice (Sal group) (Figure 1A). We found that prenatally VPA-exposed mice displayed deficits in social interaction in the three-chamber assay (Figures 1B–D). During the habituation period, both two group mice had no preference for two empty cages (Figure 1B). Once a stranger mouse was introduced into one chamber, control group mice spent more time in the mouse-containing chamber and exhibited more reciprocal social interaction time than in the empty chamber and socialized more frequently with the stranger mouse. In contrast, VPA group mice showed no preference for the stranger mouse (Figures 1C,D). When a second stranger mouse was introduced into the empty chamber, VPA group mice still showed no preference for the new stranger mouse while control group mice socialized more frequently with the new stranger mouse (Figures 1E,F). VPA-exposed mice also displayed anxiety-like behavior in the EPM test (Figures 1G,H), impaired recognition memory in NOR (Figure 1I) and repetitive behaviors in the marble-burying test (Figure 1K) and self-grooming assay (Figure 1L) without affecting locomotor activity in the OFT (Figure 1J) and attack behavior (Figure 1M), compared with saline group mice. The above results indicated that prenatally VPA-exposed mice exhibited autistic-like behaviors.

We then examined if paired-housing with normal control mice have an effects on autistic-like behaviors of prenatally VPA-exposed mice. An independent cohort of VPA and Sal animals aged at P21 were weaned in cages containing two prenatally VPA-exposed mice (VPA–PA group), two prenatally saline-exposed mice (Sal–Sal group) or one prenatally VPA-exposed mice paired with one prenatally saline-exposed mice (VPA-Sal group) (Figure 2A). After 4 weeks paired-housing began at P21 (P21–P49), we evaluated autistic-related behaviors among these group mice. We found that four-week paired-housing with saline-exposed mice ameliorated the deficits in social interaction in the three-chamber assay in prenatally VPA-exposed mice (Figures 2B–F). Furthermore, the paired-housing treatment normalized the anxiety levels in the EPM (Figures 2G,H), enhanced recognition memory in NOR (Figure 2I) and repetitive behaviors in the marble-burying test (Figure 2K) and self-grooming assay (Figure 2L) in prenatally VPA-exposed mice, while prenatally VPA-exposed mice paired-housing with the similar VPA-exposed mice still exhibited autistic-like behaviors in these tests as before (Figures 2B–L). The above treatment exerted no effect on locomotor activity in the OFT (Figure 2H) and attack behavior (Figure 2M). These results indicated that the autistic-like behaviors in prenatally VPA-exposed mice were attenuated by paired-housing with control mice.

### 3.2 The effects of paired-housing on autistic-like behaviors occur only during a critical period

To determine when pairing-housing influences autistic-like behaviors most, the prenatally VPA-exposed mice were paired-housed with different period and determined autistic-like behaviors. After one-week paired-housing with saline-exposed mice (P21–P28, Figure 3A), the prenatally VPA-exposed mice still exhibited autistic-like behaviors, with deficits in social interaction in the three-chamber assay (Figures 3B–F), anxiety-like behavior in the EPM test (Figures 3G,H), impaired recognition memory in the NOR (Figure 3I) and repetitive behaviors in the marble-burying test (Figure 3K) and self-grooming assay (Figure 3L) without affecting locomotor activity in the OFT (Figure 3H) and attack behavior (Figure 3M). However, when paired-housing with saline-exposed mice after 2 weeks (P21–P35, Figure 4A), the prenatally VPA-exposed mice exhibited ameliorated autistic-like behaviors in these behavior tests (Figures 4B–M), results similar with that in prenatally VPA-exposed mice four-week paired-housing with saline-exposed mice (P21–P49) (Supplementary Figure S1).

Furthermore, we wonder whether P21–P35 is a critical period for the influence of paired-housing on autistic-like behaviors. When paired-housing was initiated after P35 and lasted 2 weeks (Figure 5A), autistic-like behaviors were indistinguishable from those in VPA–PA mice (Figures 5B–M). Also, if prenatally VPA-exposed mice were paired-housed with saline-exposed mice from P28 to P35, autistic-like behaviors were still exist (Figures 6A–M). Thus, paired-housed with saline-exposed mice from P21 to P35 ameliorated autistic-like behaviors in prenatally VPA-exposed mice, which suggest a link between the quality of paired-housing established during the critical period and attenuation of autistic-like behaviors.

### 3.3 Normalized GABA level and GABA receptors expression in prenatally VPA-exposed mice that paired-housing during P21–P35

Our previous study found a reduced GABA level and downregulation of GABA receptors in prenatally VPA-exposed mice (Yang et al., 2021). To further determine the effects of paired-housing on these changes in prenatally VPA-exposed mice, we analyzed the levels of neuromodulators and GABA receptors in the mPFC of prenatally VPA-exposed mice that paired-housing with control mice during P21–P49 using *in vivo* microdialysis and qPCR (Figure 7A). Consistent with previous results, microdialysis analysis revealed decreased levels of GABA but not glutamate and monoamine concentration in the cerebrospinal fluid from the mPFC of VPA–PA group mice, compared with Sal–Sal group mice (Figures 7B–D). And paired-housing with saline-exposed mice after 4 weeks (P21–P49) restored GABA levels (Figure 7D). Meanwhile, we investigated the expression of four GABA-A receptor subunits and two GABA-B receptor subunits. We observed significant reductions in GABA-A receptor α1 (GABRA1), GABA-A receptor α2 (GABRA2) and GABA-A receptorβ3 (GABRB3) mRNA level (Figure 7E) and largely reduction in GABA-B receptor 1 (GABBR1) and GABA-B receptor 2 (GABBR2) mRNA level (Figure 7F), without affecting the GABA-A receptor α3 (GABRA3) (Figure 7E) in mPFC from VPA–PA group mice, which was consistent with our previous results (Yang et al., 2021). However, paired-housing with saline-exposed mice after 4 weeks (P21–P49) restored changes in these receptors (Figures 7E,F). The above results were also observed in the mPFC of prenatally VPA-exposed mice that paired-housing with control mice during P21–P35 (Figures 7G–J).

## 4 Discussion

The major findings of the present study were as follows. First, paired-housing with control mice for 4 week attenuated autistic-like behaviors in prenatally VPA-exposed mice, as assessed by the social interaction ability in the three-chamber assay and repetitive behaviors in the self-grooming assay and marble-burying test. Second, the effects of paired-housing on autistic-like behaviors occur only during a critical period. Third, paired-housing during a critical period normalized GABA level and GABA receptors expression in prenatally VPA-exposed mice. Altogether, our results suggest a critical period for paired-housing-dependent autistic behaviors and disturbed GABA system attenuation in a prenatal VPA-induced mouse model of autism.

Autism spectrum disorder (ASD) is a neurodevelopmental disorder characterized by impaired social interaction and repetitive behavior (First, 2013). The former is characterized by deficits in social–emotional reciprocity, nonverbal communicative behaviors, and deficits in developing, maintaining and understanding relationships. The latter is characterized by stereotyped or repetitive motor movements, speech, or use of objects, insistence on sameness, highly restricted or fixated interests, and hyper- or hypo-reactivity to sensory inputs (Gvozdjakova et al., 2014; Neumeyer et al., 2019; Matheson and Eichen, 2018; Wang et al., 2021; Mousain-Bosc et al., 2006; McClain et al., 2017; van der Heijden et al., 2018). Epidemiological studies performed by the Centers for Disease Control and Prevention (CDC) conclude ASD presents with a frequency of 1 in every 59 children, with males four times more likely than females to be diagnosed with ASD (Maenner et al., 2020). The etiology of the disorder remains a topic of debate—epigenetic, genetic and environmental factors are all thought to play a role (Nuzzo et al., 2020; De Felice et al., 2015; De Felice et al., 2016; Chao et al., 2020; Meyza and Blanchard, 2017).

Some intervention treatments, administered both in-person and online, have shown the ability to ameliorate symptoms in ASD patients. In one experiment, personalized online therapy led to improvements in learning, memory, anxiety, attention span, motor skills, eating, sleeping, sensory processing, self-awareness, communication, social skills, and mood (Aronoff et al., 2016). Early intensive behavioral intervention (EIBI) administered to autistic children at a young age has been shown to improve social behavior (Waters et al., 2020). Another recent meta-analysis suggests that cognitive behavioral therapy (CBT) may be useful for reduction of anxiety in some ASD presentations (Perihan et al., 2020). These examples of human intervention successes underscore the potential benefit of developing analogous treatments in animal models to elucidate mechanisms underlying ASD treatments. In animals, some studies also found that environmental enrichment improves metabolic and behavioral health in the BTBR mouse model of autism (Queen et al., 2020; Kim et al., 2019; Makinodan et al., 2017; Lacaria et al., 2012; Burrows et al., 2020; Yang et al., 2007; Yang et al., 2011), which suggest that environmental intervention was an effective form of ameliorating core and associated symptoms of ASD. Our result found that the autistic-like behaviors in prenatally VPA-exposed mice were attenuated by paired-housing with control mice, which provide a new strategy for ASD treatment.

A large body of literature suggests that intervention treatments are valuable tools to ameliorate the symptoms of ASD in human patients (Aronoff et al., 2016; Waters et al., 2020; Nahmias et al., 2019; Fuller and Kaiser, 2020; Fuller et al., 2020). And the timing of intervention treatments is important for researchers and clinicians to consider. Because early ASD diagnosis and intervention are thought to be of clinical importance (Fernell et al., 2013), in this 17-week-long experiment, mice were placed in environmental enrichment (EE) as juveniles (4–5 weeks old) and exposure to EE was maintained through adulthood. In a shorter pilot experiment, mice were placed in EE as young adults (6–10 weeks old). These results suggest that EE provides benefits when initiated at juvenile and young adult ages. Experience-based synaptic development and plasticity and its behavioral sequences occur only during critical time windows (Hensch, 2005) and early intervention has been emphasized to rescue the behavioral abnormalities in previous animal studies (Lonetti et al., 2010; Oddi et al., 2015; Makinodan et al., 2012) as well as in children with ASD (Zwaigenbaum et al., 2015). Our results found that paired-housing with saline-exposed mice from P21 to P35 ameliorated autistic-like behaviors in prenatally VPA-exposed mice. These effects persist even when mice are re-exposed to VPA-exposed mice, which suggest a link between the quality of paired-housing established during the critical period and attenuation of autistic-like behaviors.

There is increasing neuropathological and neurochemical evidence that the GABAergic system has major involvement in ASD. From postmortem tissue analysis, it has been long known that there is a decrease in the number of GABAergic cerebellar Purkinje cells in many individuals with autism (Bailey et al., 1998; Williams et al., 1980; Whitney et al., 2008; Bauman and Kemper, 1985; Ritvo et al., 1986). In the last decade, biochemical studies have revealed that there are highly significantly decreased levels of key synthesizing enzymes for GABA, glutamic acid decarboxylase type 65 and 67 (GAD65 and GAD67 isoforms) in the cerebellum and parietal cortex in autism (Fatemi et al., 2014) further suggesting that there are effects on the GABA system in the autistic brain. So disruption of inhibitory circuits might be responsible for ASD. Recent studies in animal models also demonstrate that the molecular basis of such disruption is linked to specific defects in the development and function of interneurons. Indeed, in some animal models of ASD, disruption of GABAergic circuits might underlie the emergence of autism-like symptoms, in which inhibitory neurons contain lower than normal levels of GABA (Marin, 2012). So GABAergic dysfunction has an important role in the aetiology of autism. In our study, using the prenatally exposed to valproic acid (VPA) to establish a validated ASD mouse model, we also found a reduced GABA level in these ASD mice, which was consistent with the opinion that GABAergic dysfunction was important for ASD. Meanwhile, several clinical studies has demonstrated significant reductions in protein for seven GABAA and two GABAB receptor subunit proteins in brains of subjects with autism (Fatemi et al., 2014; Fatemi et al., 2009; Fatemi et al., 2009). And our previous study similarly found a reduction of GABA receptors level in ASD animal model (Yang et al., 2021). Altogether, reduced GABA/GABA receptor levels may contribute for the development of ASD. In this paper, we also found that group housing at a critical period rescued the reduced GABA/GABA receptor levels, suggesting that this intervention has beneficial effects on ASD.

Alterations of monoaminergic neurotransmitter systems have been detected in many brain regions such as the hippocampus, the midbrain, the cerebellum, the cortex, as well as in the peripheral system such as the serum in ASD pathophysiology (Kuo and Liu, 2022). And the monoamine-related drugs are the available medications for treating ASD. Considering this, we determined the monoamine content in prenatally VPA-exposed ASD mice. The results indicated that, except the GABA level, the monoamine content was not affect, which was consistent with our previous study (Yang et al., 2021). The discrepancy of the monoamine content between our result and other studies may be that, we observed the mPFC region that exhibited no change of the monoamine content. So future study will investigate the relationship between monoamine content and ASD.

In conclusion, paired-housing with control mice palliated sociability deficits, anxiety and repetitive behaviors in the animal model of ASD-like behaviors induced by prenatal VPA exposure, and P21–P35 is a critical period for the influence of paired-housing on autistic-like behaviors and impaired GABA system, demonstrating the therapeutic potential of environmental intervention during a critical period in the treatment of ASD.

## Data Availability

The original contributions presented in the study are included in the article/Supplementary material, further inquiries can be directed to the corresponding author.
