# Supplementary figures and images for: A critical period for paired-housing-dependent autistic-like behaviors attenuation in a prenatal valproic acid-induced male mouse model of autism
Source: Front Neurosci. 2025 Jan 17;18:1467047. doi: 10.3389/fnins.2024.1467047 (PMC11782243; doi:10.3389/fnins.2024.1467047)

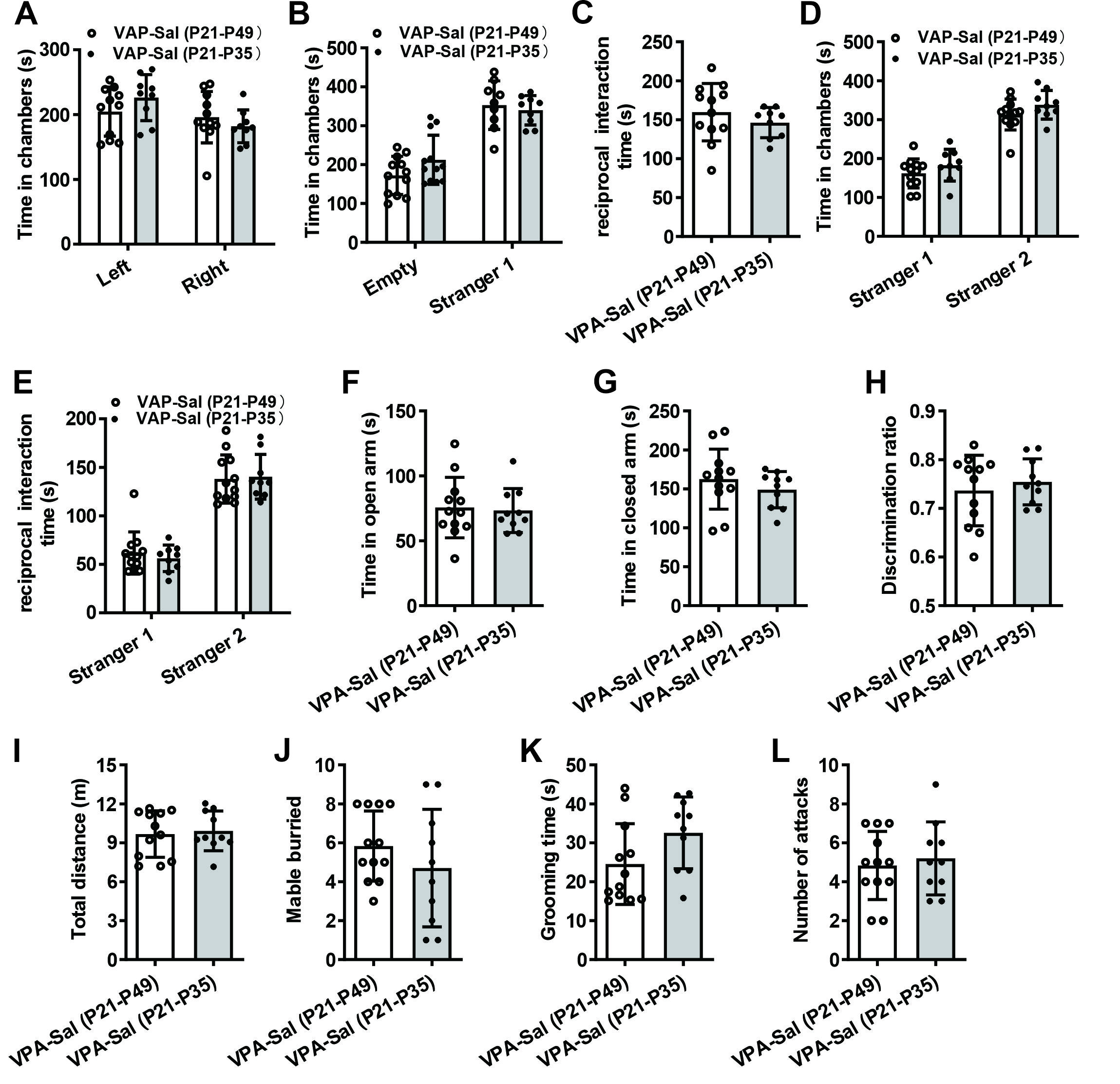

Supplement: Supplementary file 1 [file Image_1.jpg]
